# Loop-Mediated Isothermal Amplification as Point-of-Care Testing for *EGFR*-Mutated Lung Adenocarcinoma

**DOI:** 10.3390/mi13060897

**Published:** 2022-06-06

**Authors:** Yuichi Saito, Atsuka Matsui, Satoru Michiyuki, Hiroaki Morooka, Takayuki Ibi, Yoshikane Yamauchi, Nobumasa Takahashi, Yoshihiko Shimizu, Tomohiko Ikeya, Eishin Hoshi, Yukinori Sakao, Masafumi Kawamura

**Affiliations:** 1 Department of Surgery, Teikyo University School of Medicine, 2-11-1 Kaga, Itabashi-ku 173-8605, Tokyo, Japan; yoshikaney@med.teikyo-u.ac.jp (Y.Y.); ysakao070@gmail.com (Y.S.); mkawamur@med.teikyo-u.ac.jp (M.K.); 2 Department of Thoracic Surgery, Saitama Cardiovascular and Respiratory Center, 1696 Itai, Kumagaya 360-0197, Saitama, Japan; morook00@gmail.com (H.M.); t-ibi@nms.ac.jp (T.I.); ntakahas030417@gmail.com (N.T.); ikeya.tomohiko@pref.saitama.lg.jp (T.I.); eishinh1129@gmail.com (E.H.); 3 Biochemical Research Laboratory II, Eiken Chemical Co., Ltd., 1381-3 Shimoishigami, Otawara-shi 324-0036, Tochigi, Japan; atsuka_matsui@eiken.co.jp (A.M.); satoru_michiyuki@eiken.co.jp (S.M.); 4 Department of Pathology, Saitama Cardiovascular and Respiratory Center, 1696 Itai, Kumagaya 360-0197, Saitama, Japan; yshimizu1957@gmail.com

**Keywords:** lung cancer, liquid biopsy, epidermal growth factor receptor, loop-mediated isothermal amplification, polymerase chain reaction, point-of-care testing

## Abstract

Liquid biopsy has been adapted as a diagnostic test for *EGFR* mutations in patients with advanced or metastatic non-small cell lung cancer (NSCLC). Loop-mediated isothermal amplification (LAMP) has been widely used for the rapid detection of pathogens through DNA amplification. This study investigated the efficacy of an *EGFR*-LAMP assay using plasma samples of patients with resected NSCLC tumors. The *EGFR* status was investigated using both LAMP and next-generation sequencing (NGS) assays in cases that met the following criteria: (1) pulmonary adenocarcinoma with *EGFR* mutation detected by the Therascreen *EGFR* PCR Kit and (2) preoperative plasma samples contained enough DNA for the LAMP and NGS experiments. Among 51 specimens from patients with *EGFR*-mutated tumors or metastatic lymph nodes, the LAMP assay detected 1 *EGFR* mutation that was also detected in the NGS assay. However, a plasma sample that demonstrated *EGFR* wild type in the LAMP assay showed an *EGFR* mutant status in NGS. The detection rates (1.9% in LAMP and 3.9% in NGS) were very low in both assays, demonstrating a similar performance in detecting *EGFR* mutations in NSCLC tumors; therefore, it could be a more suitable test for the advanced stage, not the early stage. Notably, the LAMP assay was more time-saving, cost-effective, and straightforward. However, further investigation is required to develop a more sensitive assay.

## 1. Introduction

Lung cancer remained the leading cause of cancer death and the second most frequently diagnosed cancer in the GLOBOCAN 2020 database [1]. The 5-year survival rate after lung cancer diagnosis was only 10% to 20% in most countries from 2010 to 2014, although these rates were higher in Japan (33%), Israel (27%), and Korea (25%) [2]. In the last two decades, advances in lung cancer therapeutics have led to the adaptation of comprehensive molecular profiling of novel driver mutations, and the epidermal growth factor receptor (EGFR) tyrosine kinase inhibitors (TKIs) have been shown to improve overall response rate and median progression-free survival when compared with platinum-based chemotherapy in patients with advanced or metastatic non-small cell lung cancer (NSCLC) harboring activating *EGFR* mutations [3,4,5,6,7,8,9,10]. Thus, *EGFR* TKIs are established effective therapies in patients with activating and sensitizing mutation in exons 18–21 of *EGFR* [11,12,13,14].

Recently, NCCN guidelines noted that osimertinib, a third-generation TKI, should be considered an adjuvant treatment even after complete resection of NSCLC [14]. Until recently, osimertinib was used only as a second-line treatment for those with T790M mutations [12,13]. However, Wu et al. reported that osimertinib demonstrated statistically longer disease-free survival in patients with stage IB to IIIA *EGFR*-mutated NSCLC than in those who received a placebo [15]. Based on published evidence regarding osimertinib use in the double-blind, phase III ADAURA clinical trials, routine molecular biomarker testing of *EGFR* mutation is now recommended not only for patients with advanced or metastatic NSCLC but also for patients with completely resected stage IB-IIIA NSCLC according to the NCCN guidelines [14].

These clinical trials have called for new or revised recommendations in the molecular testing guidelines for patients with NSCLC [16,17,18,19,20,21]. Currently, several assays are accepted as *EGFR* testing methods based on the technologies of real-time polymerase chain reaction (RT-PCR) or next-generation sequencing (NGS). The cobas^®^ *EGFR* Mutation Test v2 (Hoffman-La Roche Ltd., Basel, Switzerland) and the Therascreen *EGFR* Rotor-Gene Q (RGQ) PCR Kit^®^ (Qiagen, K.K., Tokyo, Japan) were approved as companion diagnostics with TKIs by the United States Food and Drug Administration and the Ministry of Health, Labour and Welfare in Japan [18]. Similarly, FoundationOne^®^ CDx (Foundation Medicine Inc., Cambridge, MA, USA) and Oncomine^TM^ Dx Target Test (Thermo Fisher Scientific Inc., Waltham, MA, USA) were adapted for *EGFR* screening for patients with advanced or metastatic NSCLC. However, these methods have been notably complicated, time-consuming, and expensive in clinical practice.

Loop-mediated isothermal amplification (LAMP) is an alternative PCR-based technology that has been widely used globally in bacteriology [22,23,24,25] and virology, including for SARS-CoV-2 [26,27,28,29,30,31]. For instance, TB-LAMP has been used extensively, with multiple meta-analyses reporting sensitivity and specificity in the range of 89–93% and 94–95%, respectively [25,32,33]. In contrast to RT-PCR, LAMP uses 4–6 primers, does not require an expensive thermocycler, and has the potential to decrease testing cost and time to diagnosis in laboratory or community settings. Thus, the LAMP assay has been recommended in guidelines [34,35], and point-of-care diagnostics for tuberculosis have been developed with a turnaround time of about 30 min and a cost of about 7 euros per test [36]. In our previous study, 117 NSCLC tumor tissues were investigated to compare the results of the *EGFR*-LAMP and Therascreen assays. The receiver operating characteristics curve analysis of the *EGFR*-LAMP assay for LAMP values demonstrated 0.973 (95% CI: 0.929–1.00) in exon 19 deletion, and 0.952 (95% CI: 0.885–1.00) in L858R [37]. The sensitivity and specificity were 89.3% and 98.9%, respectively. Although the LAMP assay is not yet widely used in clinical oncology, it could provide a more rapid, simple, and inexpensive method for detecting oncogenes than conventional methods.

Recently, many researchers have been investigating liquid biopsy as a promising non-invasive technology for the early detection of cancer as well as continuous monitoring of disease progression and/or treatment efficacy [38,39,40,41]. In this study, we aimed to develop a new liquid biopsy system using *EGFR*-LAMP primers for NSCLC. We compared findings between the LAMP and NGS assays and evaluated their sensitivity and specificity.

## 2. Materials and Methods

### 2.1. Study Design

**Figure 1:**
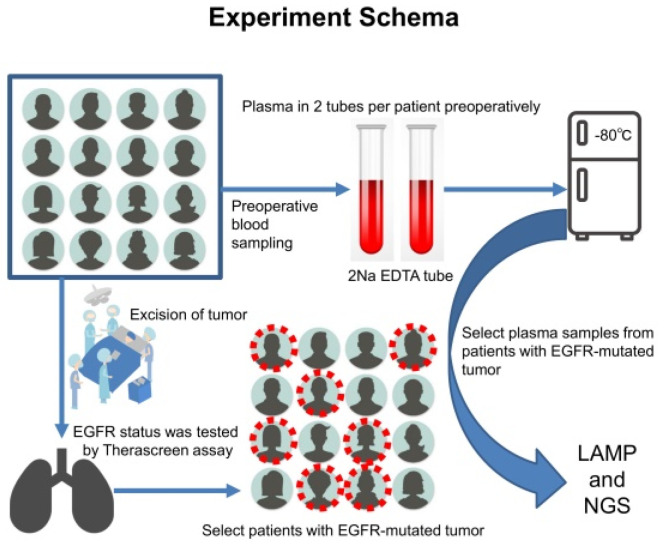
Experimental schema of this study. Plasma samples were obtained from patients with a strong suspicion of primary lung cancer and stored in a freezing chamber preoperatively. After surgery or surgical biopsy, all tissue samples were fixed with 10% buffer formalin at 20–25 °C (24–36 h) to create formalin-fixed, paraffin-embedded tumor blocks. Afterward, hematoxylin–eosin-stained slides were prepared for pathological diagnosis. The Therascreen assay was performed to investigate the *EGFR* status of resected tumor tissues from select cases with *EGFR*-mutated adenocarcinoma. DNA extraction was performed from plasma samples for both the LAMP and NGS assays.

This study was a prospective study without interventions to investigate the sensitivity and specificity of the *EGFR*-LAMP liquid biopsy compared with those of the NGS assay. The objects of this study were patients who had surgical biopsy or treatment for NSCLC suspected at Saitama Cardiovascular and Respiratory Center (Saitama, Japan) from January 2019 to September 2020. Clinical data and plasma samples were collected from patients preoperatively and consecutively, and tumor tissue samples were obtained through surgery or surgical biopsy (Figure 1). Any other pulmonary diseases without NSCLC were excluded from this study. Only *EGFR*-mutated pulmonary adenocarcinomas detected using the Therascreen assay were selected for further investigation for liquid biopsy. The present study was approved by The Institutional Review Board of the Saitama Cardiovascular and Respiratory Center (approval no. 2018033). Written informed consent was provided by all patients. The study was conducted in accordance with the Declaration of Helsinki (as revised in 2013). There were 3 primary endpoints for the study: (i) confirmation of the detection of *EGFR* mutations in NSCLC using the *EGFR*-LAMP primers, (ii) calculation of sensitivity and specificity, and (iii) comparison with results of the NGS assay. We planned to collect 50 *EGFR*-mutated tumors to increase the probability of detecting at least one *EGFR* mutation in the LAMP assay.

### 2.2. Plasma Samples and DNA Extraction

Blood samples were obtained preoperatively and consecutively from patients with a strong suspicion of primary lung cancer. Through normal centrifugation (4 °C, 3000 rpm, 15 min) within 8 h from blood sampling, plasma separation was performed by sampling from the center of the plasma supernatant. Among these, postoperative pathology demonstrated 57 *EGFR*-mutated primary adenocarcinomas considered eligible for this study. After excluding 6 patients who did not provide written informed consent, cell-free DNA (cfDNA) from plasma samples was extracted using the cobas^®^ cfDNA Sample Preparation Kit (Roche Diagnostics, Hague Road, IN, USA) according to the manufacturer’s protocol.

### 2.3. Pathological Diagnosis and Therascreen qPCR Mutation Analysis

All tumor tissues were obtained through surgery or surgical biopsy and fixed with 10% buffer formalin at 20–25 °C (24–36 h) to create formalin-fixed, paraffin-embedded tumor blocks according to the recommendation of The Japanese Society of Pathology [42]. Hematoxylin–eosin (HE) staining was performed by the standard methods using Tissue-Tek Prisma^®^ (Sakura Finetek Japan Co., Ltd., Tokyo, Japan) according to the manufacturer’s protocol. All pathological diagnoses were made based on the WHO classification version 8 by an expert pulmonary pathologist who examined HE-stained slides in low and high magnification using an ECLIPSE Ni-u light microscope (Nikon Co., Ltd., Tokyo, Japan). The presence of *EGFR* mutations was determined using a Therascreen *EGFR* PCR kit^®^ (Qiagen, K.K.) [43].

### 2.4. LAMP Mutation Analysis

**Figure 2:**
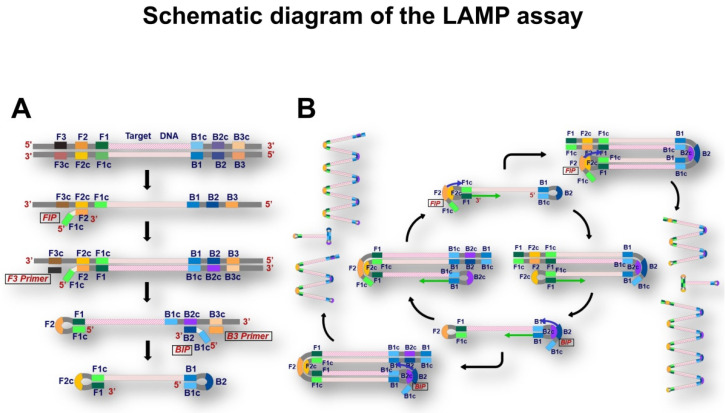
Schematic diagram of loop-mediated isothermal amplification. (**A**) Scheme of dumbbell-like structure formation. Target DNA is firstly denatured into single-stranded DNA. Then, the forward inner primer (FIP) initiates DNA synthesis from the F2c region. The following extension from the forward outer primer (F3) displaces the strand that was generated by the FIP extension. The replaced strand plays the role of a template of the backward inner primer (BIP) and backward outer primer (B3) in the same manner as sense strand amplification. Consequently, the dumbbell-like structure harboring hairpin structure on both of 5′- and 3′-terminus is generated. (**B**) Scheme of isothermal amplification. The dumbbell-like structure exposing F2c or B2c regions where FIP or BIP hybridizes initiates cycling amplification. The cycling amplification regenerates dumbbell-like structures and generates various amplicons, which are exponentially increased in length.

The LAMP assay was performed according to the standard protocol described in our previous studies [37,44]. Briefly, the LAMP assay was conducted at 65 °C for 120 min using the LightCycler 480^®^ (Roche Diagnostics KK, Tokyo, Japan) and a primer set that was developed in our laboratory (Figure 2). After denaturing the amplicon at 95 °C for 5 min followed by hybridization at 37 °C for 5 min, the temperature was gradually raised to 80 °C, and the fluorescence intensity was measured 7 times per 1 °C increment., The data were analyzed using the LightCycler 480 software^®^ (version 1.5.1.62; Roche Diagnostics KK) to calculate the melting peak.

### 2.5. Next-Generation Sequencing

Amplicon-based NGS was performed using the MiSeq system (Illumina KK, Tokyo, Japan), and the primer sets for amplification of *EGFR* exons 18, 19, 20, and 21 were used as previously reported [37,40]. The resulting FASTQ files were mapped to the GRCh38 human reference sequence using BWA-MEM (http://bio-bwa.sourceforge.net/ (accessed on 5 June 2022)). The variant data was extracted from the mapped data using Samtools ver. 1.9 (http://www.htslib.org/ (accessed on 5 June 2022)) and GATK4 (https://gatk.broadinstitute.org/hc/en-us (accessed on 5 June 2022)).

### 2.6. Statistical Analysis

Descriptive statistics and categorical variables were calculated using standard formulae with Excel 2019 ver. 16.0.12527.20260^®^ (Microsoft Corp, Tokyo, Japan) and SPSS Statistics^®^ version 28 (IBM Corp., Armonk, NY, USA). The sensitivity and specificity of the LAMP and the NGS assays were obtained, and figures were created using Excel 2019 and the statistical software R version 3.6.3 (R Foundation for Statistical Computing, Vienna, Austria) with the ggplot2 package. Clinical and pathological findings were extracted from the electronic medical records of the patients. *p* < 0.05 was considered significant.

## 3. Results

### 3.1. Examination of the Efficacy of *EGFR*-LAMP Liquid Biopsy

**Figure 3:**
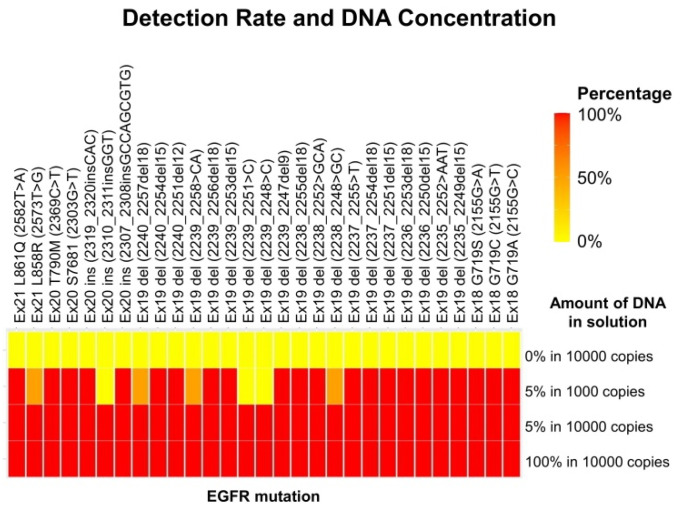
The relationship between results of the LAMP assay and DNA concentrations. The *x*-axis shows 4 concentrations, and *Y*-axis presents 29 subtypes of *EGFR* mutations. The LAMP assay detected all *EGFR* mutations over 5% in 10,000 copies.

To investigate the performance of *EGFR*-LAMP liquid biopsy in detecting *EGFR* mutations, four kinds of solutions with different concentrations of mutated *EGFR* copies (0%, 5%, and 100% in 10,000 copies and 5% in 1000 copies) were prepared in the preliminary study (Figure 3). The detection of *EGFR* mutations was marked by 29 *EGFR*-LAMP primers in a solution of 5% in 10,000 copies. Detection was achieved in 26 primers but not in three primers. Thus, a concentration of at least 5% in 10,000 copies may be required to detect *EGFR* mutations using the LAMP assay.

### 3.2. Characteristics of Patients with *EGFR*-Mutated Adenocarcinoma

A total of 264 plasma sampling and surgical procedures were performed as a biopsy or treatment for patients with highly suspicious lung cancer. Among them, 57 resected tumors demonstrated primary lung cancer with *EGFR* mutations, which was confirmed by the Therascreen assay using tumor tissues. After excluding patients without informed consent, 51 patients were enrolled in this study. The mean age of the 51 patients was 68.7 years (49–85 years), and most patients were female (n = 31, 70.8%) (Table 1). There were 22 never-smokers (43.1%), 23 former-smokers (45.1%), and 6 current smokers (11.8%). In terms of histologic subtype of adenocarcinoma, there were 45 papillary predominant (88.0%), 4 lepidic (8.0%), 1 micropapillary (2.0%), and solid predominant (2.0%) adenocarcinomas. Most cases were pathological stage I, consisting of 12 p-IA1 (24.0%), 21 p-IA2 (41.0%), and 6 p-IA3 tumors (12.0%).

### 3.3. Amount of cfDNA Extracted from Plasma Samples

**Figure 4:**
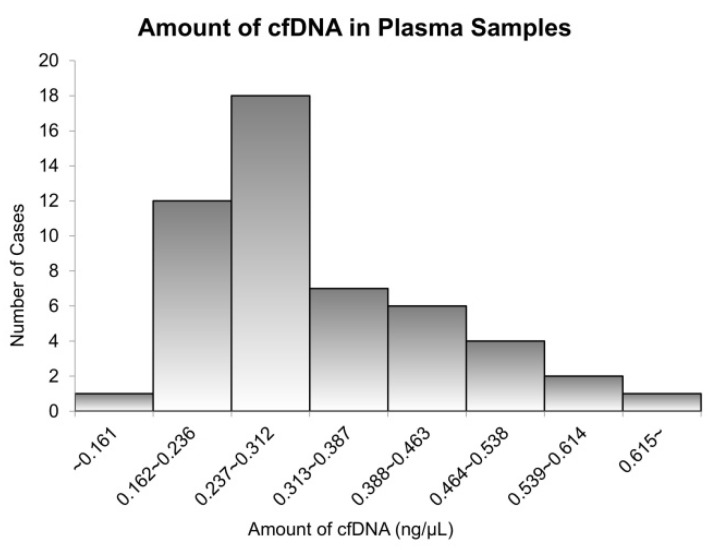
The relationship between the number of cases and cell-free DNA (cfDNA) concentration. The *x*-axis shows various concentrations of cfDNA, and *Y*-axis presents the number of cases. The median concentration of cfDNA extracted from plasma samples was 45.92 ng/μL, the minimum concentration was 25.76 ng/μL, and the maximum concentration was 110.24 ng/μL.

The cfDNA was obtained from clinical plasma samples using the QIAamp DNA Blood Mini Kit^®^ (Qiagen, K.K.). Figure 4 illustrates the amount of cfDNA extracted from each specimen. In extracted cfDNA, the average value was 50.89 ng (25.76–110.24), and over 0.162 ng/μL cfDNA was available for use in most cases (Supplementary table). Among them, the quantity of cfDNA that we actually used was 1.59 ng (0.81–3.45) on average for the LAMP assay and 5.54 ng (2.83–10.00) for the NGS assay. Therefore, the cfDNA volume was considered enough for both the LAMP and NGS assays.

### 3.4. Therascreen EGFR PCR Mutation Analysis for Resected or Biopsied Tumors

Among the 51 adenocarcinomas, there were 27 tumors with exon 19 deletion (52.9%), 18 tumors with exon 21 L858R point mutations (35.3%), 2 tumors with exon 20 in-frame insertion (3.9%), 2 tumors with exon 19 deletion/exon 20 in-frame insertion (double mutation) (3.9%), and 2 uncommon *EGFR* mutations (exon 19 deletion/G719X point mutation and G719X/exon 18 point mutation) (Table 2).

### 3.5. Comparison of Results of the LAMP and the NGS Assays

Among the 51 tumors, there was one tumor with *EGFR* mutations in both the LAMP and the NGS assays, another one with *EGFR* wild type in the LAMP assay but *EGFR* mutations in the NGS assay, and the others with *EGFR* wild type in both assays. Both the LAMP and the NGS assays were not able to detect *EGFR* mutations in 49 plasma samples, even though the mutation was confirmed through the Therascreen assay in the resected or biopsied tumors. Among two cases with *EGFR* mutations in the NGS assay, one case had p-stage IVB adenocarcinoma with an L858R point mutation in the LAMP assay, while the other had p-stage IIIA adenocarcinoma with an L858R point mutation only in the NGS assay (Table 3). In the supplementary table, the amount of cfDNA was 1.44 ng in the LAMP assay detecting *EGFR* mutation (average 1.59 ng in all LAMP experiments), and it was 5.04 ng and 6.58 ng in the NGS assay resulting in *EGFR* mutations (average 5.54 ng in all NGS experiments). A relationship was not found between the amount of cf DNA and the detection of *EGFR* mutation in both assays. The positivity rate of the *EGFR*-LAMP liquid biopsy was 2.0%, and that of the NGS assay was 3.9%.

## 4. Discussion

The identification of *EGFR* mutations is necessary for decision-making in the treatment of patients with advanced or metastatic NSCLC. Furthermore, the latest NCCN guidelines recommend testing *EGFR* status even after complete resection for patients with early-stage NSCLC to consider the administration of adjuvant chemotherapy [16,17,18,45]. Due to the expanded indications for *EGFR* examination, the demand for a rapid and cheap approach to identifying *EGFR* status has been steadily increasing. However, health insurance only allows testing through conventional PCR-based or NGS-based methods in most countries. Since the latter method is time-consuming, expensive, and highly complex, there is an urgent need to develop a more convenient approach for molecular testing of oncogenes.

Point-of-care testing (POCT) is a concept where testing for healthcare is provided close to or near the patients. Although various definitions have been provided in the medical or scientific field, it was defined as “testing performed near or at the site of a patient with the result leading to a possible change in the care of the patient” [46]. POCT is being increasingly used in general practice inside and outside the hospital, such as in the case of the reverse transcription LAMP (RT-LAMP) for COVID-19 [47]. The standard for COVID-19 diagnostics are PCR-based tests that are highly sensitive, specific, and remarkably reliable, but these tests are limited by the requirement for sophisticated laboratories and skilled personnel, the complex protocol, the long waiting times for results, and an overall high cost per test. The development of RT-LAMP can be a robust solution for the rapid and cost-effective diagnosis of COVID-19 in low-resource laboratory settings. Although basic research into POCT for several diseases has been progressing drastically, there are only a few reports of POCT in oncology for now.

In 2018, Asaka et al. reported a POCT system for detecting *EGFR* mutations based on a droplet-polymerase chain reaction (d-PCR) assay using cfDNA from patients with lung cancer [48]. The *EGFR* d-PCR assay was designed to detect three *EGFR* mutations: L858R in exon 21, E746_A750del in exon 19, and T790M in exon 20. However, it has not been adopted in clinical practice yet. In this manner, the development of POCT is just beginning in oncology, and it is widely expected that POCT will conquer some technical limitations of conventional methods.

In this pioneering study, the *EGFR*-LAMP assay successfully detected *EGFR* mutations using 29 *EGFR*-LAMP primers developed and reported in our previous study [37,44]. However, an *EGFR* mutation was only detected in one case. The low positivity rate for *EGFR* mutations is a clinical issue in the development of the *EGFR*-LAMP liquid biopsy. The reason for this is likely not the limits of abilities of the LAMP assay but the low concentration of circulating tumor DNA (ctDNA) because the NGS assay also demonstrated a low positivity rate. The ctDNA concentration could be below 5% in 1000 copies according to the relationship between the detection rate and DNA concentration (Figure 3). Consequently, using plasma samples from patients with advanced or metastatic tumors instead of early-stage tumors is recommended to confirm the positivity rate of *EGFR* mutations in *EGFR*-LAMP liquid biopsy. Another improvement will be the development of a multiplex platform equipped with multiple *EGFR*-LAMP primers, allowing us to avoid the subdivision of plasma samples for each primer. However, it is necessary to elucidate the optimal combinations of *EGFR*-LAMP primers to allow accurate gene amplification.

Currently, the cobas *EGFR* assay^®^ (Roche Molecular Systems Inc., CA, USA) for analysis of plasma samples has been officially validated in NSCLC by authorized organizations. However, it is inconvenient because of the long waiting time to obtain results. Despite the limitations of this study (small number of cases and study participants from a single institute), we were able to demonstrate the potential of *EGFR*-LAMP liquid biopsy as POCT in oncology as well as in the general field of molecular testing. In future studies, we plan to investigate this application by using plasma samples from patients with more advanced-stage tumors.

This study had several limitations: (1) relatively small sample size, (2) single-institute nature of the study, (3) data were obtained only from surgery cases, and (4) negative control groups (tumors with *EGFR* wild type) were missing. Since ctDNA concentration could be high in plasma samples from more advanced-stage tumors in theory, further studies should be planned to clarify the performance of the *EGFR*-LAMP liquid biopsy system for advanced cancers through comparisons with negative control groups.

In conclusion, our study was the first to demonstrate that the LAMP assay successfully detected *EGFR* mutations in plasma samples, illuminating a direction for the development of POCT in oncology. POCT of *EGFR* mutations could dramatically change clinical practice in the future. For instance, an outpatient’s blood could be examined, and the result of the oncogene status could be obtained within half a day. This could also be applied to staging in place of the TNM classification, assessment of the effects of chemoradiation, and elucidation of resistance to anticancer drugs.

## Figures and Tables

**Table 1:** Clinical characteristics of patients in the study.

Characteristic	N (%)
Age, years	68.7 ± 8.4
Sex	
Male	20 (29.2)
Female	31 (70.8)
Smoking Status	
Never smoker	22 (43.1)
Former smoker	23 (45.1)
Current smoker	6 (11.8)
Subtype of adenocarcinoma	
Papillary predominant	45 (88.0)
Lepidic predominant	4 (8.0)
Micropapillary predominant	1 (2.0)
Solid predominant	1 (2.0)
Pathological stage	
pIA1	12 (24.0)
pIA2	21 (41.0)
pIA3	6 (12.0)
pIB	2 (4.0)
pIIB	3 (6.0)
pIIIA	4 (8.0)
pIIIB	1 (2.0)
pIVA	1 (2.0)
pIVB	1 (2.0)

Data on age: mean ± SD.

**Table 2:** *EGFR* status of resected tumor tissue as determined using the Therascreen assay.

	Number	Percentage (%)
Del 19	27	52.9
L858R	18	35.3
Ins 20	2	3.9
Ex 19 del + Ins 20	2	3.9
Del 19 + G719X	1	2.0
G719X + Ex 18	1	2.0
Total	51	100

*EGFR*, epidermal growth factor receptor; Del 19, Exon 19 deletion; Ex 18, Exon 18 point mutation; Ins 20, Exon 20 insertion; L858R, Exon 21 L858R point mutation.

**Table 3:** Comparison of *EGFR* status between resected tumor and preoperative plasma samples.

		Tumor		Plasma	
	p-Stage	Therascreen	LAMP	LAMP	NGS
Case 1	IVB	L858R	L858R	L858R	L858R
Case 2	IIIA	L858R	negative	negative	L858R

*EGFR*, epidermal growth factor receptor; L858R, Exon 21 L858R point mutation; NGS, next-generation sequencing; LAMP, loop-mediated isothermal amplification.

## Data Availability

The data used to support the findings of this study are included within the article.

## Supplementary Materials

The following supporting information can be downloaded at: https://www.mdpi.com/article/10.3390/mi13060897/s1, Table S1: Amount of cfDNA.
